# AI-powered digital probing improves periodontal disease diagnosis

**DOI:** 10.1016/j.xcrm.2025.102185

**Published:** 2025-06-17

**Authors:** Jiamin Wu, James Kit Hon Tsoi

**Affiliations:** 1 Dental Materials Science, Faculty of Dentistry, The University of Hong Kong, Hong Kong SAR, P.R. China; 2 Clinical AI Research Theme, Faculty of Dentistry, The University of Hong Kong, Hong Kong SAR, P.R. China

## Abstract

Current periodontal disease diagnosis often relies on manual probing. Here, Tan et al. present PerioAI, an AI system combining intra-oral scans and cone-beam CT for automated, precise, and non-invasive periodontal assessment, enhancing early detection and guiding treatment without manual probing.

## Main text

Periodontal disease affects hundreds of millions worldwide, causing irreversible gum and bone damage that leads to tooth loss. Nearly 800 million people suffer from severe periodontitis, often undetected until advanced stages, complicating treatment and prognosis.^1^^,^^2^^,^^3^ Early and accurate diagnosis is critical for effective intervention. Traditional diagnosis relies on manual periodontal probing, which measures periodontal pocket depth (PPD) for decades but is limited by variability in clinician technique, patient discomfort, and sparse sampling sites.^4^ These factors reduce accuracy and may deter regular assessments.

Digital imaging and AI offer promising alternatives.^4^^,^^5^^,^^6^ Panoramic radiographs provide 2D overviews but suffer from distortion and cannot accurately assess 3D bone defects or PPD. More advanced 3D technologies like intra-oral scans (IOS) and cone-beam computed tomography (CBCT) deliver detailed soft tissue and bone images, aiding visualization and surgical planning.^7^^,^^8^ However, these tools focus on specific diagnostic aspects and require manual measurements. Integrating soft tissue inflammation data with bone loss visualization remains challenging, making treatment planning complex and prone to variability among clinicians.

In a significant advance published in *Cell Reports Medicine*, Tan et al.^9^ present PerioAI, a pioneering digital system for automated periodontal disease diagnosis. PerioAI integrates multimodal dental data (i.e., IOS and CBCT images) to deliver a novel, comprehensive and quantitative assessment method for periodontal health. This AI-driven system not only emulates aspects of the clinical workflow by directly calculating the gingiva-bone distance (GBD)—a critical parameter reflecting the true extent of periodontal tissue destruction—but also presents clear visual information on soft tissue morphology from IOS and bone loss severity from CBCT.

The PerioAI system provides a comprehensive full-stack pipeline comprising four sophisticated components. First, an IOS segmentation module accurately identifies and delineates dental crowns. Concurrently, a CBCT image segmentation module extracts precise models of the teeth and alveolar bone. Third, a multimodal data fusion module aligns the segmented crowns from IOS data with the corresponding teeth from the CBCT image. This accurate registration by integrating the soft and hard tissues information is paramount for the following diagnosis. Finally, a digital probing measurement module infers the gingival and alveolar bone contours along the longitudinal axes of each tooth, enabling automated GBD calculation, in theory, at any number of points. Based on these GBD measurements and predetermined classification thresholds, PerioAI delivers tooth-level, visualized, and true 3D volumetric diagnostic outcomes.

The power of PerioAI lies in its ability to overcome many limitations of traditional periodontal diagnostic methods. By automating the measurement process, it significantly reduces operator-dependent variability, resulting in more reproducible and objective diagnoses. The non-invasive nature of digital image acquisition, compared to physical probing, enhances patient comfort and compliance. Moreover, by analyzing the complete gingiva-tooth-bone relationship from the fused 3D data, PerioAI can provide a far more detailed and comprehensive 3D visualized assessment than the standard six-point manual probing, potentially identifying early or localized disease foci that might otherwise be missed. Although the system relies on imaging devices such as IOS and CBCT, it is relatively common in today’s hospitals and clinics and is usually used as a routine examination method for patients undergoing wisdom tooth extraction, restoration, orthodontics, implants, and other treatments.

PerioAI was trained and validated on an extensive multicenter dataset involving 2,507 patients, demonstrating remarkable performance. Tan et al.^9^ evaluated their multimodal segmentation and registration algorithms separately, achieving superior and robust accuracy in both internal and external datasets. Furthermore, the digital probing measurements were evaluated in both digital and clinical contexts. Specifically, in the digital evaluation phase, the digital probing method measures the GBD on 360 points from the previous AI-aligned results compared to optimally segmented and aligned results by dentists, with an exceptionally low error margin of 0.040 mm. In addition, digital probing simulates clinical probing in periodontal disease diagnosis by measuring six sites, resulting in a clinical probing comparable solution, especially in the early stage of periodontal disease.

The implications of PerioAI are significant and far-reaching. For clinicians, it promises to streamline clinical diagnostic workflows, reduce cognitive load, and offer extensive quantitative and visual data to support precise treatment planning, particularly for complex dental cases requiring regenerative or resective therapies. Moreover, the ability to accurately map GBD across the dentition can guide targeted interventions and allow for more precise monitoring of treatment outcomes. For patients, this technology could translate into earlier detection of periodontal disease, reduced discomfort during examinations, and ultimately better preservation of natural dentition.

Looking ahead, AI systems like PerioAI could become integral to routine parts of dental check-ups, facilitating widespread early-stage screening and enabling population-level studies on periodontal disease progression. Future developments might include integrating PerioAI with longitudinal patient data to track disease activity over time, predict individual treatment responses, guide robotic-assisted periodontal therapies, and serve as an integral part of therapy with, e.g., true 3D generative-AI restoration system.^10^ Further validation in diverse patient populations and real-world clinical settings will be essential, subject to regulatory bodies’ approval and integration into existing dental software platforms.

In conclusion, the work by Tan et al.^9^ on PerioAI represents a landmark advancement in dental diagnostics. By seamless fusion of multimodal imaging data through a sophisticated AI-driven diagnostic pipeline, PerioAI offers a powerful, automated, and non-invasive tool for diagnosing periodontal disease. This innovation not only addresses long-standing limitations in the field but also paves the way for a new era of precision and personalized dental care, holding the promise of significantly improving oral and general health outcomes worldwide.

## Declaration of interests

The authors declare no competing interests.
